# On the Characterization and Software Implementation of General Protein Lattice Models

**DOI:** 10.1371/journal.pone.0059504

**Published:** 2013-03-29

**Authors:** Alessio Bechini

**Affiliations:** Department of Information Engineering, University of Pisa, Pisa, Italy; Wake Forest University, United States of America

## Abstract

Abstract models of proteins have been widely used as a practical means to computationally investigate general properties of the system. In *lattice models* any sterically feasible conformation is represented as a self-avoiding walk on a lattice, and residue types are limited in number. So far, only two- or three-dimensional lattices have been used. The inspection of the neighborhood of alpha carbons in the core of real proteins reveals that also lattices with higher coordination numbers, possibly in higher dimensional spaces, can be adopted. In this paper, a new general parametric lattice model for simplified protein conformations is proposed and investigated. It is shown how the supporting software can be consistently designed to let algorithms that operate on protein structures be implemented in a lattice-agnostic way. The necessary theoretical foundations are developed and organically presented, pinpointing the role of the concept of *main directions* in lattice-agnostic model handling. Subsequently, the model features across dimensions and lattice types are explored in tests performed on benchmark protein sequences, using a Python implementation. Simulations give insights on the use of square and triangular lattices in a range of dimensions. The trend of potential minimum for sequences of different lengths, varying the lattice dimension, is uncovered. Moreover, an extensive quantitative characterization of the usage of the so-called “move types” is reported for the first time. The proposed general framework for the development of lattice models is simple yet complete, and an object-oriented architecture can be proficiently employed for the supporting software, by designing ad-hoc classes. The proposed framework represents a new general viewpoint that potentially subsumes a number of solutions previously studied. The adoption of the described model pushes to look at protein structure issues from a more general and essential perspective, making computational investigations over simplified models more straightforward as well.

## Introduction

In the wide assortment of reduced models of proteins [1], minimalist representations have been proposed and widely used in the last decades as a practical means to computationally investigate general properties of these polymers [2], [3]. In pursuing simplification, the number of possible conformations can be reduced by imposing residues to be located only on vertices of a given lattice. According to this vision, in the so-called *lattice models*, any valid (i.e., sterically feasible) conformation is represented as a self-avoiding walk on the lattice [4], with adjacent residues placed on adjacent vertices. Moreover, the number of residue types can be drastically restricted, e.g. just setting apart “hydrophobic” and “polar” ones, as it happens in the *HP model*, which can be regarded as the paradigmatic example [5] of protein lattice models. The HP model was originally proposed on a square two-dimensional lattice and it has also been exploited to understand the behavior of specific real proteins, such as chaperonins [6], [7].

Generally speaking, lattice models have found application in multiple aspects of theoretical investigations on proteins: exploration of the conformation space [8], analysis of folding pathways [9], dynamics and thermodynamics of the folding process [10], evolutionary issues and origin of long-range interactions [11], strategies to enforce protein stability [12]. Although the large majority of lattice models do not encompass a representation of side chains, they are able to accommodate also this possibility and in some studies a single residue is modeled with a lattice point for the alpha carbon along with an adjacent point for the whole side chain [13], [14]. Recently, lattice models have been used to characterize the placement of termini in native protein structures [15], and they have found application also in the investigation of RNA folding [16]. For many applications, the accuracy gap between all-atom models and lattice models is indeed significant, and attempts to bridge it have been proposed by projecting the former onto the latter via optimization methods [17], [18].

Despite their simplicity, lattice models actually show protein-like features [13], indicating that they incorporate the fundamental physical principles of proteins.

### General Lattice Models: How and Why

In the context of the present discussion, the primary structure of a protein can be represented with a string 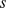 whose characters are taken out of a standard alphabet 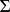 that encodes the possible monomer types. E.g., in the classical HP model we have 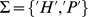. The symbol 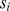 (or 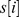) indicates the type (character) of the 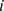-th residue.

For the sake of clearness and precision, a lattice 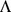 is defined as a subset of 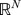, containing *vertices* (points) that are orderly placed. In the most regular case, it can be expressed as
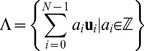
(1)


The set of vectors 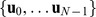 is a basis for 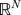 that, respect to the standard basis, can be represented by the basis matrix 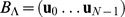. Any lattice vertex is identified through its integer coordinates 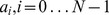. Moreover, it is sometimes useful to explicitly consider also *edges* in the lattices, i.e., the unordered couples of vertices that represents “connections”. In a large number of practical applications, a reasonable way to specify edges makes use of a given threshold for the distance of vertices that possibly define an edge:
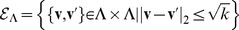
(2)using the ordinary euclidean norm 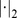. The value of 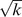 is generally set to the minimum yielding 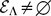. Regardless of the way edges are specified, two distinct vertices in an edge are said to be *adjacent*.

Protein lattice models have been proposed so far on different lattice types, in two and three dimensions. An extensive formal treatment of lattices is beyond the scope of this discussion: further details can be found e.g. in the book by Conway and Sloane [19]. In this paper, a generalization of conformation studies by employing parametrical regular lattices is proposed, along with indications on how software support can be provided to this aim. In this perspective, the lattice type and dimension can be simply regarded as two parameters in the characterization of the search space. This approach is motivated by the observation that, in a lattice model, the neighborhood of each residue placed on a given vertex corresponds to the set of adjacent vertices, which are necessarily limited in number. More formally, in a lattice the *coordination number* (here indicated with 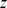) is the number of adjacent vertices for any single vertex (i.e., the cardinality of the so-called *vertex neighborhood*), and it depends both on the lattice type and dimension. Thus, we can accommodate as many other residues around any given residue as the coordination number of the used lattice. A viable way to increase this number is just scaling up the lattice dimension. This has been currently done passing from 2D to 3D models, but the process can be further extended. A caveat is mandatory: High dimensional models, although possibly actractive for several aspects, may lead to conformations with an unnatural surface/volume ratio, hampering their employment in the study of solvent interactions, or of multichain systems.

In the core of real proteins, residues are densely packed and alpha carbons are close to one another. We can quantitatively characterize such an accommodation, and subsequently we can to check whether one specific lattice type, with its own coordination number, is able to correctly describe the neighborhood of an alpha-carbon (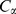). To this extent, the radial distribution of 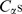 can be investigated. By 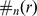 we indicate the number of other 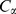
s included in a sphere of radius 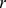 centered on a given 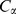. Such an investigation is significant for residues in the central part of a globular protein, for 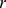 values corresponding to a sphere completely embedded in the molecule. The symbol 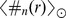 denotes the average 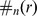 calculated for 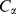
s around the molecule centroid, within an upper bound 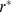 (e.g. 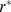 can be taken as 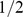 of the molecule gyration radius).

**Figure 1:**
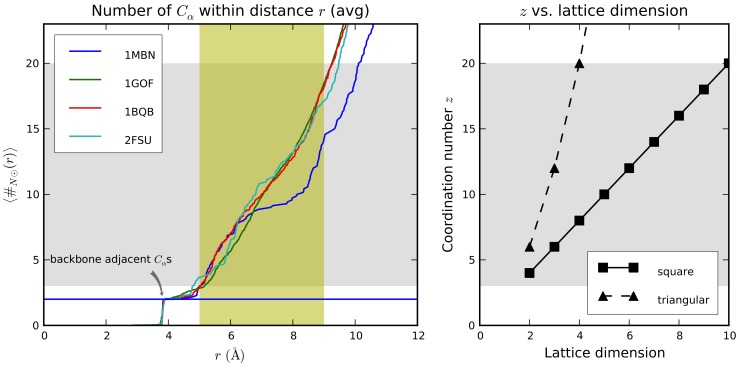
Characterization of the 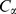 neighborhood in terms of number of other 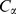
s within distance 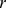. In the left chart, the curves correspond to four sample proteins of different classes, and the *admissible range* is pointed out; in the right chart, lattices falling in such an interval can be spotted.

Each protein structure has its own actual shape of 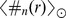, but similar patterns for low values of 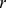 can be found even across different structural classes of globular proteins. In the first chart of Figure 1, four sample molecules from different classes (all-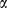, all-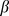, 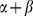, 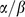), identified in PDB as 1MBN, 1GOF, 1BQB, 2FSU are considered. The shapes of their 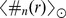 are very similar up to 7 Å, and then slightly diverge. Choosing other proteins, the specific shapes may be different, but the overall trends are analogous. A simple analysis of the reported curves can suggest how many “neighbors” a 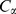 may have, depending on the defined neighborhood is defined. Usually, in studies on the interactions among residues, it is assumed that the maximum distance for interacting residues is 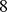 Å (in any case, less than 10 Å). In fact, it has been shown that this limit is sufficient to characterize the hydrophobic behavior of amino acid residues and to accommodate both the local and non-local interactions [20]. In other specific studies on contact potentials, cutoff values of 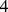, 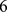, 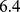, 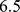, or 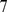 Å have been employed [21]. According with the cited results, here we assume that the neighborhood of a 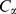 cannot reasonably extend beyond 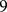 Å.

In the left chart of Figure 1, the yellow vertical belt indicates the range of possible values for the upper bound to be used in the definition of a 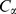 neighborhood. Thus, any lattice model that is expected to correctly represent such a neighborhood should have a coordination number not lower than 3–4 and not higher than 19–20, as shown by the shaded horizontal belt that visualize such a rough interval, hereafter called *admissible range*. Now it is possible to point out the regular lattice types whose coordination number is included in the admissible range.

The most popular lattices employed for simplified protein models since the first works in this area [2], [5] are the *square lattice* in two dimensions, and the three-dimensional *cubic lattice*, with coordination numbers 4 and 6 respectively. An unpleasant feature of this kind of lattices, known as *parity constraint*, is that no two residues can be placed on adjacent vertices if they are separated along the backbone by an odd number of residues. This limitation has pushed towards the exploration of different lattice types [22], [23]. Other significant choices are the planar *triangular lattice*, and the *FCC* (Face Centered Cubic) *lattice* (which can be considered as a thee-dimensional generalization of the former), with coordination numbers 6 and 12 respectively. Finally, also the two-dimensional *honeycomb lattice*, called also *hexagonal*, with coordination number 3 (thus at the very lower boundary of the admissible range), has been explored [24]; this one is not a Bravais lattice, and the directions to reach neighbor vertices depend on the specific vertex we are placed on.

For all the lattice types usually employed so far for simplified protein models, the coordination number is located in the lower part of the admissible range. From this standpoint, the FCC lattice represents the most appropriate choice to model the 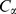 neighborhood. In order to keep the basic structure of a square lattice, to overcome the parity constraint, and to increase the number of neighbors, studies on extended cubic lattices have been proposed [14] (obtained choosing the standard basis in Eq. (1), and 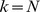 in Eq. (2)). Other exploration possibilities may easily come from the adoption of lattices in generic spatial dimensions, even beyond the classical planar and three-dimensional cases. In this work, a uniform handling of residue placement upon lattice vertices is pursued, and thus the following requirements are stated: i) all edges must have the same length, ii) all vertices must have the same coordination number, and iii) all neighbor points must be reached from any vertex by always moving in directions taken from the same given set. This last property does not hold for the planar honeycomb lattice, which cannot be generalized according to a straightforward, linear course. For this reason and taking into consideration the previous properties, the proposed generalization stems from square and triangular lattices. This does not represent a loss of generality, because the honeycomb model can be plainly obtained within the planar triangular lattice by imposing an angle of 120 degrees between any couple of subsequent bonds, and this constraint can also be added in the case of triangular lattices in higher dimensions.

The generalization of the square lattice to 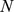 dimensions is trivial, and the dependence of the coordination number from the dimension can be expressed as 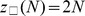. This means that, in the square case, even going up to 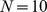, the lattice remains in the admissible range. The generalization of the triangular lattice to 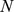 dimensions is less self-evident. In our treatment, the corresponding coordination number can be expressed as 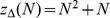, and the maximum 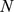 to fall in the admissible range is 4. These discussed limits for the coordination numbers can be directly visualized in the right chart of Figure 1.

In this work it has been developed a computational framework to deal with the lattice type and dimension in a seamless way, so that as a protein model is instantiated in the first place, the lattice specifications must be provided at the same time. One challenging issue is to organize the framework so that any algorithm that operates on the model may abstract as much as possible from the actual lattice details.

In the subsequent “Methods” section the theoretical tools to support generic lattice models in any dimension are presented, and it is discussed how algorithms can be accordingly developed, possibly within a neat object-oriented scheme.

In the “Results and discussion” section, by employing two classical optimization methods on benchmark sequences, the main features of the proposed models across lattice types and dimensions are uncovered, as well as the characterization of possible simple modifications to the conformations (typically used in Monte Carlo approaches).

## Methods

The generalization of lattice models requires some basic notions that will be illustrated in the following, starting from the 2D case. In the first place, it is crucial understanding how to structurally characterize the lattice model, given its type and dimensions as model parameters. Later, lattice-agnostic functions to deal with the model must be developed, as well as methods alike to manipulate conformations. The computation of the chosen potential function can thus rely on such generic functions, and possibly more efficient versions for specific values of the model parameters can be developed.

### Definitions

**Figure 2:**
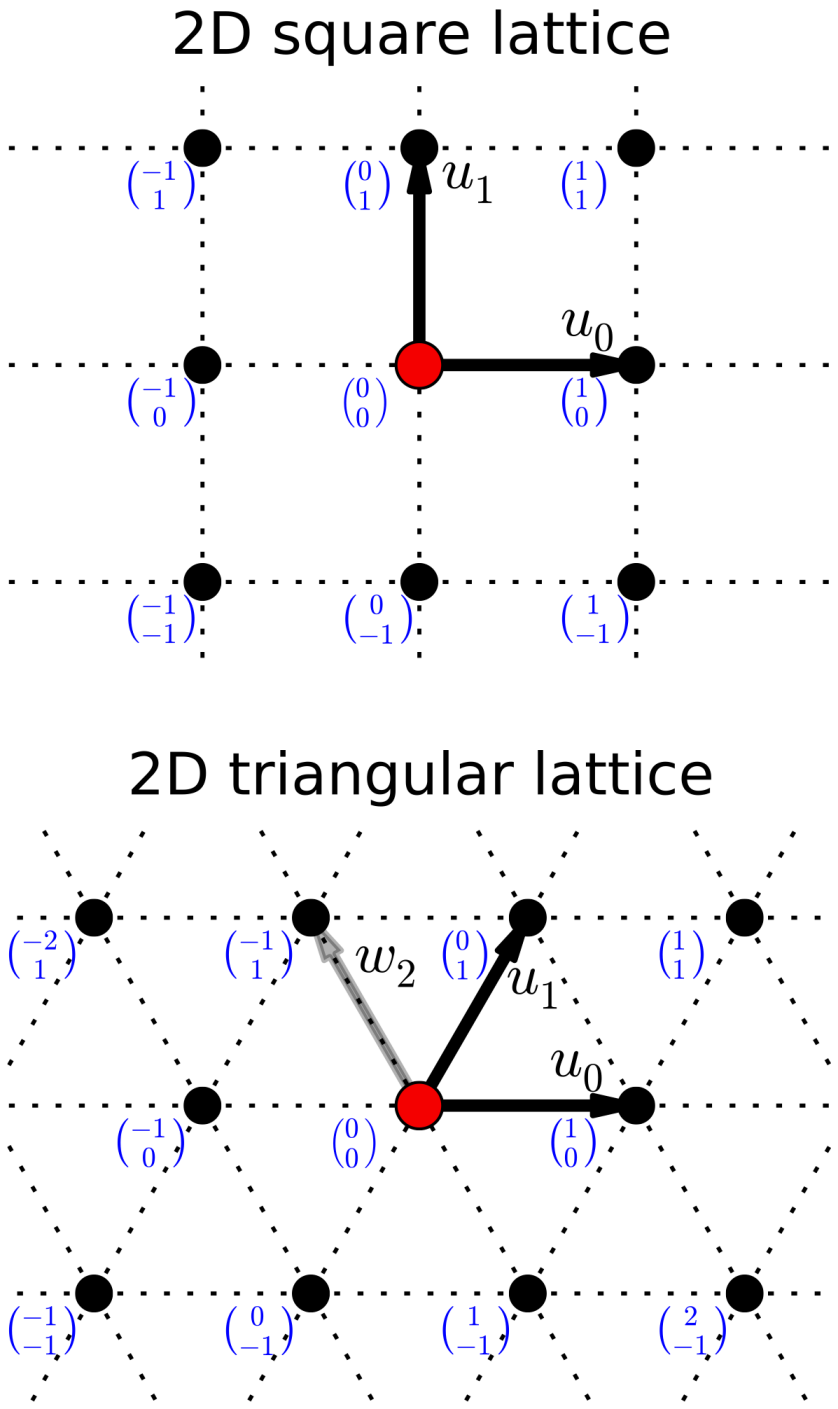
Square and triangular lattices in two dimensions, along with possible bases. Edges (relative to 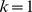) are indicated by dotted segments; the origin is marked in red.

According to the desired regularity of the employed lattices, the discussion is focused on square and triangular lattices. All the edges are imposed to have the same length that, with no loss of generality, is assumed to be 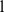. In general, different bases can be chosen for the same lattice. The basis vectors are often referred to as *primitive vectors*. In Figure 2 the two-dimensional square and triangular lattices are represented, along with the coordinates of vertices in the chosen basis (which, for the square lattice, is the ordinary standard one). From a computational standpoint, it is not always convenient to resort to the standard cartesian coordinates to locate vertices. On the contrary, the employment of the definition in Eq. 1 let us use just integer values to this purpose.

A possible proper basis for the two-dimensional triangle lattice is shown in the second chart of Figure 2, whose corresponding basis matrix (referred to the standard basis) is
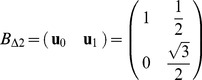
(3)


In the general case, the set of *main directions* 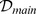 contains the (unit) vectors that, summed to the position vector of a given vertex, produce the position vectors of *all* the neighbor vertices. As previously recalled, 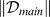 corresponds to the *coordination number* of the specific lattice. Within computations, the availability of 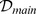 values let us check adjacency of two vertices in a straightforward way.

For the 2D square lattice (see Figure 2), 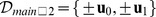. For the 2D triangular lattice, some additional considerations are needed. It is worth underlining that the position vector 
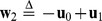
 corresponds to a neighbor of the origin, and that in this case 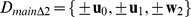. In both cases, the set of main directions can be generated by the repeated application of a rotation to 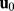. Working in the triangular lattice with the chosen basis, such a counterclockwise 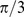 rotation is expressed by
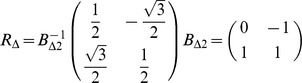
that notably contains only integer values. Making use of 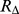, we have 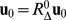, 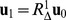, 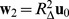, 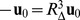, and so on. Thus, an alternative formulation for the set of main directions of the 2D triangular lattice is the following: 
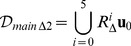
(4)


#### Scaling up to 3D and beyond

The extension of the *square* lattice up to three or more dimensions is straightforward, as the chosen basis can trivially be the standard one. The main directions correspond to 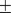 the basis vectors, so in dimension 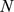


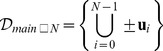
(5)and the coordination number will vary as 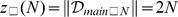


Again, the triangular case deserves more attention. In 3D, the 2D basis vectors 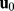 and 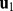 can be kept, adding 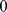 as their last coordinate; the third basis vector 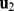 can be chosen imposing its unit length, and the value 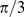 to angles 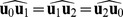, i.e., 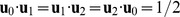. In compliance with this choice,
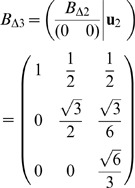
(6)


The lattice generated by such a basis 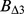 is the well-known FCC (Face Centered Cubic). Any further extension up to higher dimensions can be dealt with in the same way, iteratively building the basis 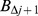 starting from the known 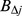 and imposing the same constraints on length and angles:
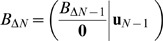


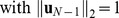
(7)

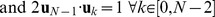



In the chosen 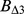 for FCC, any possible vertex adjacent to the origin can be either 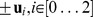 or 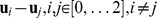.

So the main directions are calculated as 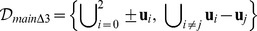, and in the general case
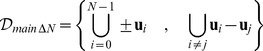
(8)


Accordingly, the coordination number depends on 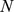 as 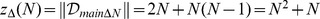, as already shown in the right section of Figure 1.

Equations (5), and (8) can be plainly used in dedicated software libraries to compute the main directions for any specified lattice. Such a set of vectors (as well as the basis matrix) can be subsequently referred and generically exploited in algorithms of any type, that operate on the lattice model, regardless of the actual dimension. For the sake of clarity, the set 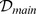 can be sorted e.g. according to a lexicographic order, and kept in a list 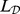, so that 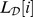 refers to the 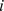-th main direction.

### Handling Conformations

By convention, the initial residue of a conformation self-avoiding walk can always be placed on the lattice origin. The most natural way to represent a conformation is by means of an *ordered* list of 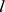 vertex coordinates in the chosen basis. Such a list is referred to as *absolute encoding* 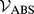, and 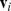 (or 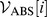) indicates its 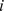 ^th^ element, 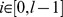. An alternative way is the *differential encoding* 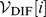, i.e., a sequence of 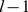 *steps* (i.e., vectors in 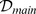) such that 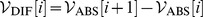.

Often, it can be useful checking whether a list of lattice vertices corresponds to a self-avoiding walk. It is required that both i) any vertex (but the first) is just one step away from the previous one, and ii) no clash is present, i.e., no position vector occurs more than once in the list. The self-avoidance check can be trivially implemented in a completely general way, given the set of main directions, because the first condition imposes that every element in 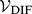 must be a vector out of 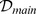, while the second condition asks just for equality checks between integer vectors of the same dimension (in 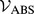). The computational complexity of the self-avoidance check procedure, intrinsically quadratic in the list length, can be made linear by using a hashtable to keep information on the occupancy state of any vertex in the lattice portion where the conformation is located [16].

In the literature, by pursuing the specification of a conformation regardless of its orientation respect to the origin, the *relative encoding* 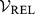 has been introduced for 2D and 3D lattices. It corresponds to an ordered list of 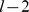 relative move types, and each relative move type specifies how to actually perform the next step, given the previous ones. In 2D, it is additionally required to fix the first move, and usually by convention also the second residue is constrained to be placed on a given vertex. The move type corresponds to the specific planar rotation 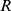 to transform a given differential move into the next. Schiemann et al. [25] explicitly discuss the case of relative moves for the 3D cubic lattice. In higher dimensions and with triangular lattices, this kind of conformation representation is not so straightforward and intuitive as in the basic cases, and for this reason it is not addressed here.

The most elementary operations to handle a configuration correspond to rigid-body transformations. Working with lattices, both translations and rotations must carry any source vertex exactly onto a destination vertex. To this aim, in any dimensions, permitted translations are expressed by a vector in 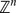, and the absolute encoding of the translated conformation can be plainly obtained by adding such vector to each element of 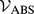. On the contrary, 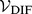 is translation-insensitive.

Rotations deserve more attention, because even if they are intuitive and familiar transformations on the plane or in 3D, they must be adequately generalized in higher spaces. A popular way to extend the ordinary concept of rigid rotation to the n-dimensional case makes use of the so-called *linear hypothesis*, i.e., the assumption that in 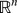, the rigid rotation is performed about an 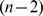-dimensional subspace. The problem is treated in a concise yet clear way by Mortari [26]. According to this assumption, it is possible to calculate rotation matrices 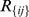 that transform one “source” main direction 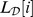 into another “destination” 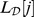, about a subspace normal to both. In general, 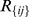 not necessarily maps 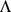 onto 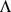, although for square lattices this happens in any dimension.

**Figure 3:**
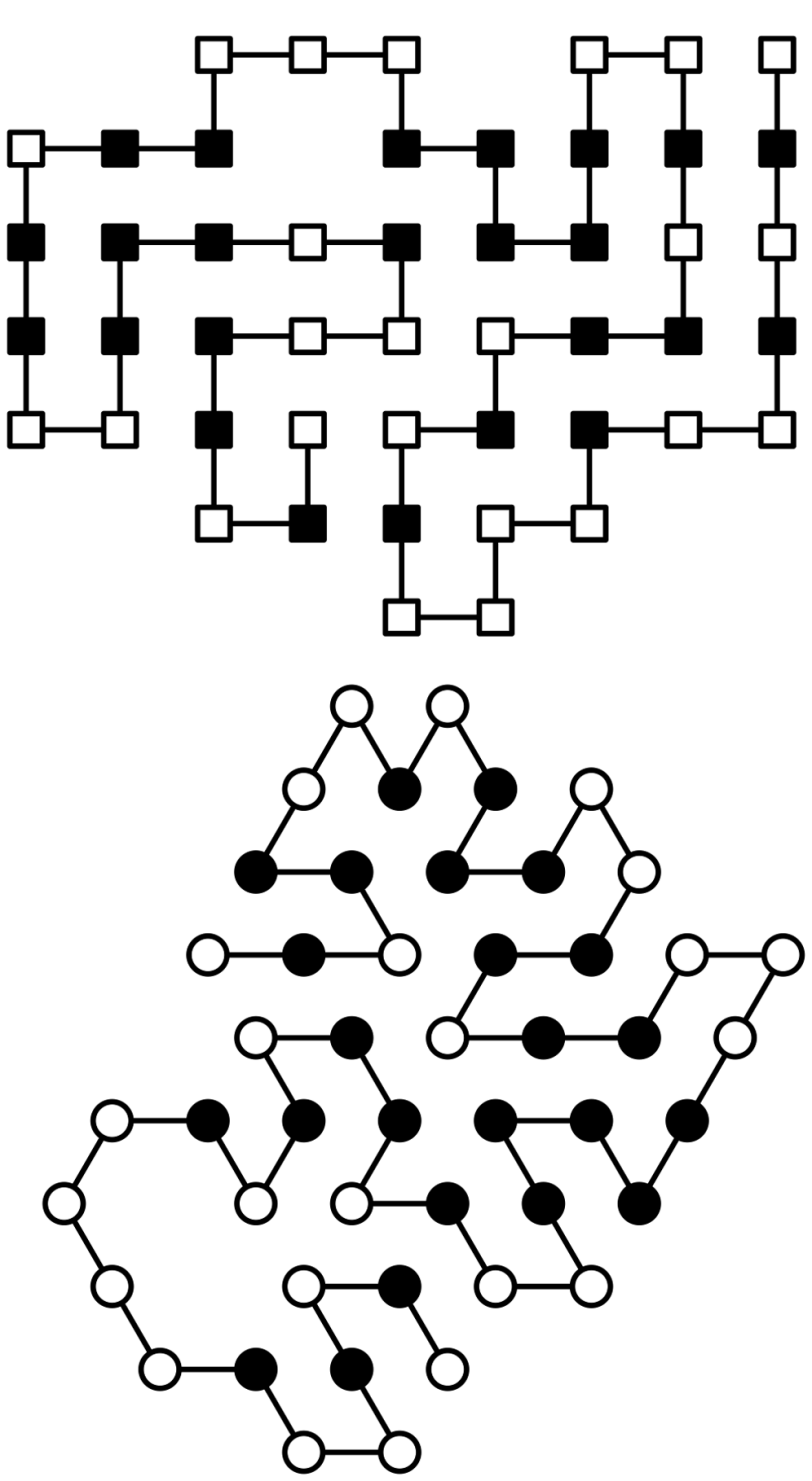
Two conformations of the same sequence (HI4) in the 2D square and triangular lattices. The HP potential is −16 for the former, and −31 for the latter.

**Figure 4:**
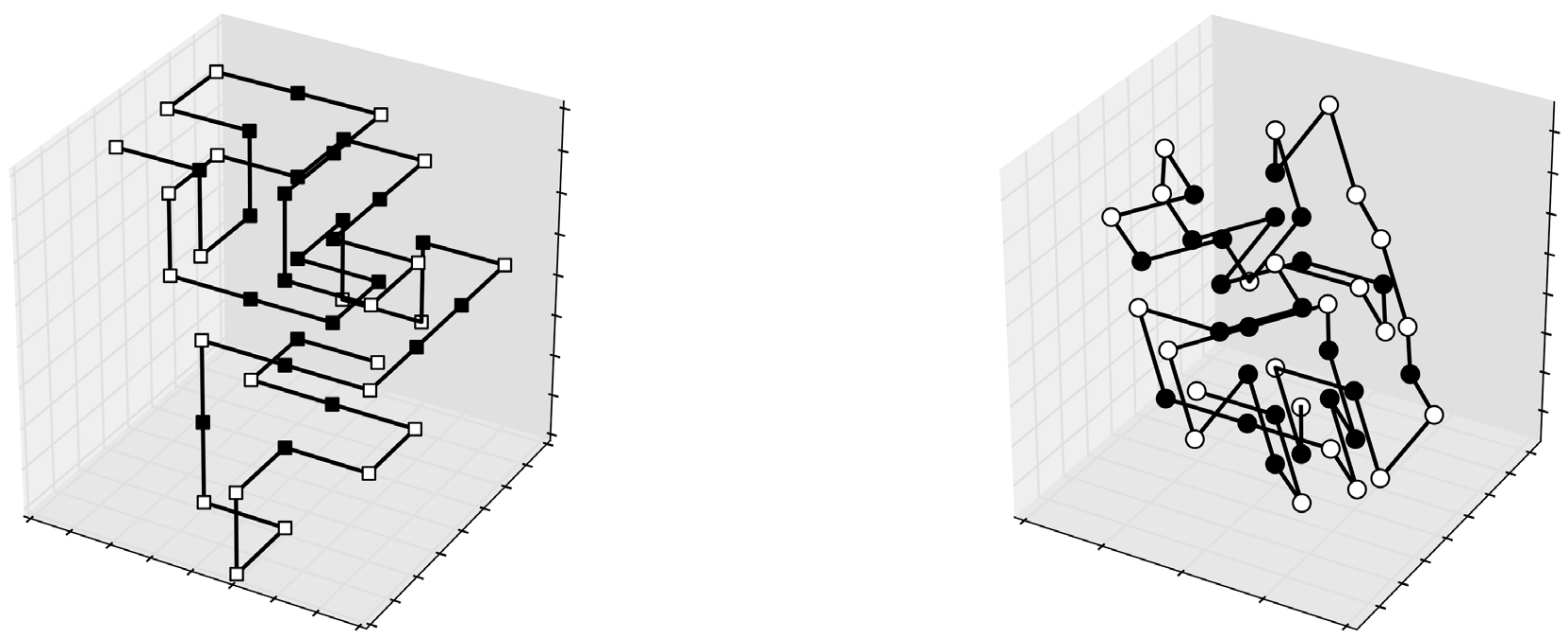
Two conformations of the same sequence (HI4) in the 3D square and triangular lattices. The potential is −27 for the former, and −60 for the latter.

Examples of 2D conformations on square and triangular lattices are depicted in Figure 3. Only H and P residue types are considered in the models, and the classic HP potential has been used for the reported values. Moreover, examples of conformations of the same sequence on 3D lattices are reported in Figure 4.

#### Distance on the lattice and computation of potential

In protein lattice models the notion of potential is usually related to some kind of *contact*, i.e., placement on adjacent vertices of residues that are not adjacent on the primary structure [27]. The adjacency of two lattice vertices, whose coordinates are expressed according to the basis matrix 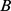, can be checked simply by inspecting if 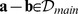. In any case, the computation of potential requires the employment of a distance function 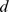 over the lattice. Considering the kind of lattices addressed in this work, for any 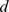 and for any couple of adjacent vertices 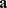, it holds 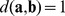. The euclidean distance can be plainly computed as 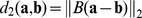.

In practical cases, often it may be more convenient adopting a notion of distance that is simpler to deal with and quicker to compute than 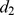. For example, the “hop distance” 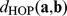 can be defined as the minimum number of hops across adjacent lattice vertices to go from 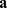 to 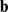. For square lattices in any dimension 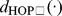 corresponds to the classical “Manhattan distance” 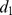, and thus it can be computed very quickly.

On triangular lattices, the computation of 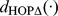 is quick in the two-dimensional case. Indicating with 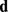 the value 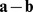, and with 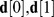 its coordinates in the basis indicated in Figure 1, the hop distance is the sum of the two smaller values among 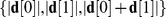. Conversely, in higher dimensions it may need a very significant amount of operations. In general, the basic idea to compute it can be sketched as follows: The value of the Manhattan distance 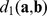 depends on the basis chosen to express the coordinates for 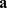 and 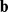; thus it must be found the specific basis 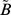, picking 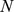 linearly independent unit vectors out of 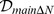, such that, indicating with 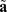 and 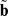 the coordinates of 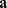 and 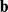 according to 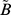, the corresponding 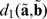 would be minimal.

In practice, to limit the computational effort, it might be sensible to have recourse to an approximate evaluation of 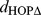 that is indeed exact for the lower values (typically for 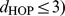 i.e., those that are involved in the definition of simplified potentials.

A fundamental component of a lattice model is the corresponding potential function, which associates a conformation with a measure of its energy. It typically contains contributions from each possible couple of residues [28], except those corresponding to adjacent residues along the backbone (in fact, their relative position is not allowed to change, and such contribution would always be constant). It is usually expressed in a form like the following:
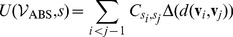
(9)


The factor 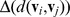 takes into account the inter-residue distance. As only neighboring residues give a significant contribution, 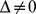 only for low values of 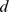. In the classic HP potential, 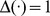 only for residues in contact (i.e., with 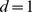). The coefficient 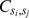 depends on the types of residues 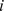 and 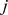. In the HP model potential, 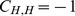, and it is 0 in all the other cases, i.e., it is the number of HH contacts times (−1). If all the different amino acids are explicitly considered in the alphabet 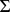, a popular choice for such coefficients is the one defined for the MJ potential [27], [29].

### Lattice-agnostic Implementation of Lattice Models and Algorithms

**Figure 5:**
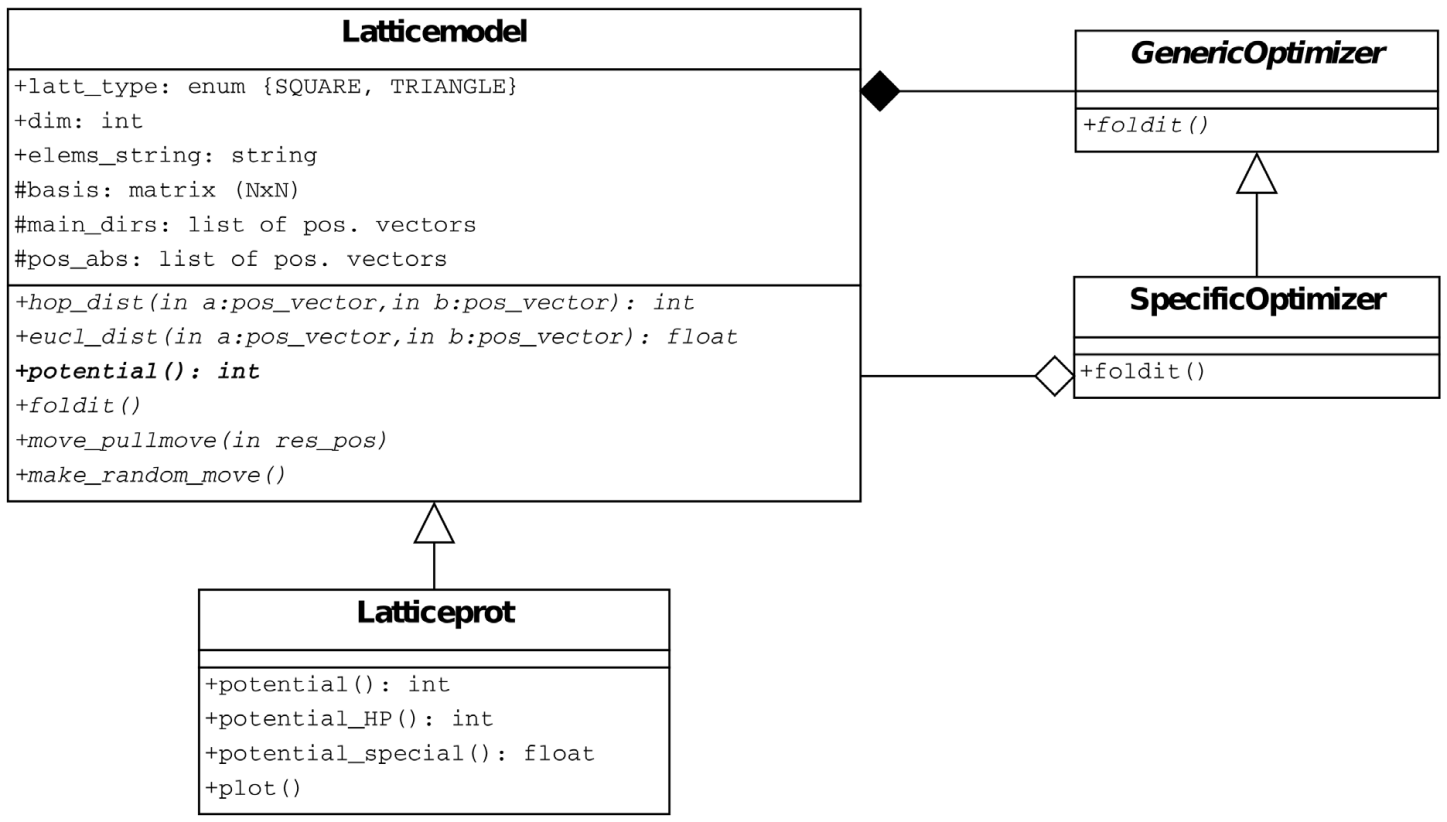
The features of generic lattice models are captured by related classes. Here an UML diagram shows the classes whose methods must be able to deal with any lattice type and dimension.

Following an object-oriented approach in developing software libraries to handle protein lattice models, the structural and behavioral details of the model can be caught by dedicated classes, as depicted in Figure 5. A class can be dedicated to lattice models in general (indicated as Latticemodel), and a derived class Latticeprot to hold features typical of protein lattice models.

The structure specification is given by both the lattice type (either “square” or “triangle”) and the dimension 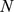. Moreover, a string with characters out of a known alphabet can be used to describe the primary structure. This information must be provided to the class constructor, so that all the supporting structural data (like the basis matrix 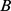, kept in the attribute basis, and 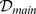, in the list main_dirs), could be computed at initialization time. Moreover, the conformation 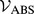 can be represented as a list pos_abs of position vectors of dimension 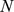 each, to be allocated by the constructor. The initial configuration is set up by the class constructor by choosing, for the position vectors in pos_abs, values that correspond to a self-avoiding walk, i.e. which satisfy the conditions previously mentioned. Multiple different build-up procedures can be provided to this aim, and the one to be used can be specified through a constructor parameter. In our implementation, the default choice is a *random walk*.

The model behavior can be encoded in methods. Among them it is worth recalling the hop distance (the 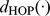 discussed before, possibly specified as class method) and the potential (to be computed upon the configuration, thus specified as instance method). It is particularly important underlining that in principle all the methods must be implemented in a lattice-agnostic way, i.e., without exploiting properties that hold only for specific lattice types or dimensions. This can be done by leveraging the structural attributes set up at the class instance initialization. Algorithm 1 (Table 1) shows a clear example: a Python *simplistic* method to obtain the HP potential. Calculations are carried out on *position vectors*, and so the dimension does not require to be explicitly considered. Here, the Numpy library [30] is used and imported as np; position vectors are implemented via Numpy arrays; the function np.dot() performs the standard vector inner product. The code can also abstract the lattice type: At line 11 (commented out), the check on the distance of two H vertices is performed just applying the definition of euclidean distance on the coordinates values, that unfortunately holds only for square lattices. Instead, such a check could be carried out as specified in line 12, by exploiting the current main directions. Of course, several kinds of improvements can be applied to improve the effectiveness of the function implementation.

**Table 1:** Algorithm 1. The HP potential can be easily obtained in a general way.

1: **def** potential(self):
2: *l* = len(self.pos_abs)
3: partial_pot = 0
4: **for** *i* **in** xrange(*l* − 2):
5: **if not** self.res_string[*i*] = = ‘H’:
6: **continue**
7: *j* = *i* + 2
8: **while** *j* < *l*:
9: **if** self.res_string[*j*] = = ‘H’:
10: 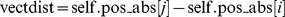
11: **# if** *np.dot(vectdist,vectdist) = = * 1*: * ***#*** * valid only for square lattices*
12: **if** **any**( (vectdist = = *v*).all() **for** *v* **in** self.main_dirs): **#** *general formulation*
13: partial_pot + = CHH **#** CHH is −1
14: *j* + = **i**
15: **return** partial_pot

It makes use of both the configuration positions and the main directions for the specific lattice.

For performance reasons, in special cases (i.e., for particular types and/or dimensions) it could be sensible to substitute the generic definition of a method with one specific efficient implementation that applies only in such cases. To this aim, depending on the programming language, different coding solutions can be found. In Python and other scripting languages, it could be up to the class constructor at initialization time to bind the generic method name to an additional method that holds the specific implementation.

**Figure 6:**
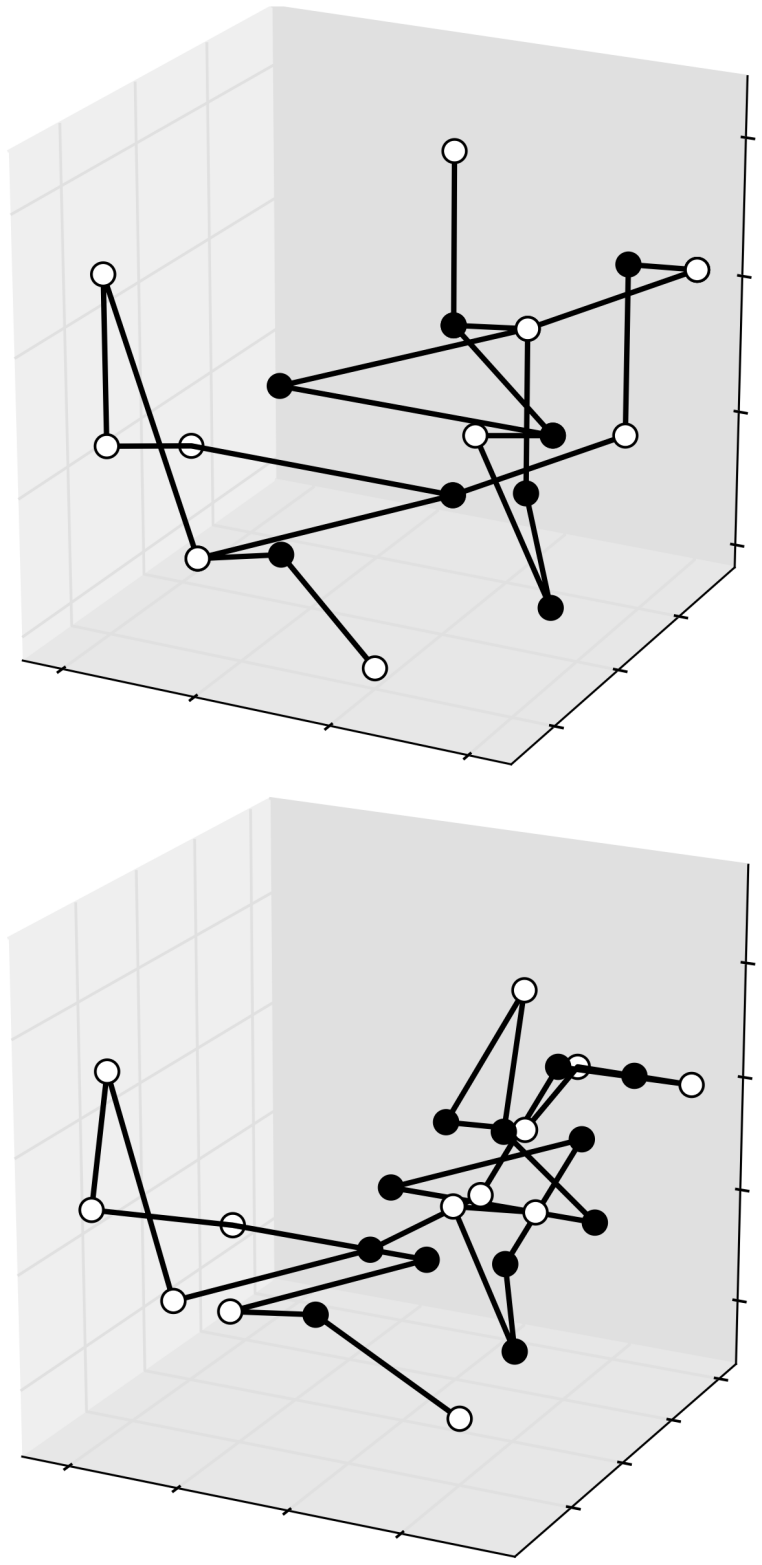
3D projections of a 5D configuration for sequence HI 9/2, in a triangular lattice. The corresponding potential is −31. Both images come from an orthogonal projection and in the second, to uncover the superposed vertices, a preliminary slight rotation in the 5D space has been applied.

A general plot() method in the Latticeprot class may represent a convenient way to visualize a conformation. Its implementation must inspect the structural model parameters in the class instance, and accordingly draw the graphical representation (typical examples of outcomes of this method are depicted in Figures 3 and 4). In case of lattices in dimension greater than 3, a representation of the projection of the conformation onto the 3D space may be used, as shown in Figure 6: only three axes amongst all are considered, possibly after a rotation of the whole structure.

The application of different folding algorithms to a given protein model can be directly supported in the Latticeprot class by recurring to the behavioral *strategy pattern* (see [31], page 315). The actual algorithm can be implemented in a separate dedicated “Optimizer” class whose instances hold all the required optimization parameters, to be provided at the object initialization. Each specific optimizer can be associated to a single protein model instance at a time, and the optimizer must be coded abstracting from the lattice characteristics: Simply, it just has to get references to the basic data structures and methods of the object it is linked to. The folding process can then be triggered by invoking a standard method on the protein model, namely foldit(), and this will (possibly) in turn call the corresponding procedure within the currently bound optimizer (if any). This approach allows us seamlessly apply different optimizators to the same protein model at different times, as well as use exactly the same optimization method on different models.

#### General moves on general lattices

Monte Carlo sampling methods have been extensively employed upon heteropolymer lattice models. Such approaches modify a conformation by applying *moves* out of a well-defined *move set*. Each particular move transforms a conformation into another admissible one [2], [32]–[34], which cannot be superposed to the former (symmetries are not considered as moves). It has been widely experimented that the effectiveness of a specific Monte Carlo approach strictly depends on the used move set [35]. One of the earliest move sets was proposed by Verdier and Stockmayer [36], and it contains only two moves that operate upon a single residue, namely the *single residue end move* and the *corner move*. Such approach was further developed by Hilhorst and Deutch [37], who also introduced the two-residue *crankshaft move*. These three move types have been used together e.g. by Gurler et al. [38]. Following Thachuk et al. [39], here they are collectively called *VSHD set*. Dealing with multidimensional models, VSHD moves have to be accordingly generalized.

The **single residue end move** can be described in a general way as the placement of an end residue onto a free vertex that is one step away from its adjacent residue, and its implementation is trivial.

The **corner move** (also known as *kink-jump move*) can be re-formulated as the placement of a target residue onto a free vertex that is one step away from both its two adjacent residues. This definition can be used (and coherently implemented) for any type of lattice in any dimension.

Similarly, although it was originally conceived for square/cubic lattices, the definition of the **two-residue crankshaft move** can be generalized as follows: Given four successive residues 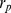, 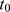, 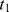, and 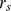 on vertices 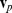, 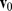, 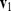, and 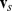, place 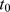 and 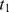 in two free vertices 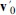 (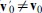) and 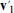 (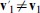) such that all the pairs (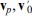), (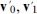), and (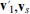) contain adjacent vertices.

The generalized VSHD moves just described can be viewed as *local* ones, as their application affects only residues in the very neighborhood of the target residue(s). On the contrary, the use of *global* moves (sometimes called also *long-range* moves [40]) generates more distant conformations. For the sake of completeness, it must be recalled that in specific studies some authors [41] have proposed moves based on breaking and reforming the chain. As they are applied for special purposes, the discussion will be focused on the generalization of global moves that keep the chain connected.

The **slithering snake move** (a.k.a. *reptation*, see [32], sect. 4.7.2) looks first for a free position adjacent to a target end, and such terminus is moved onto it. The other residues are all shifted by one position along the backbone path. This move, although it keeps most of the overall position arrangement, may completely disrupt the adjacency pattern of residues of different types, thus deeply impacting on the global potential. This definition can be taken as valid for any lattice type and dimension.

The **pivot move** (a.k.a. *wiggle*, see [32], sect. 4.7.2 or [2]) represents an effective way to drive a potentially radical change in the conformation, and it has been used in plenty of simulation works on bi- and three-dimensional lattices [2], [34], [40]. In practice, one residue 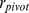 on vertex 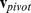 is chosen as “pivot”, and one branch departing from it, either the forward or the backward one, is wholly rotated around 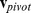 to a new position. Let 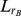 and 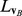 respectively indicate the lists of residues and absolute positions of the chosen branch, so that 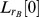 and 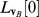 would correspond to the residue/vertex adjacent to 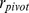 and 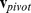, and so on. The rotation can be specified by indicating what lattice vertex 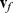, both free and adjacent to 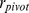, 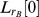 should be moved to. For the implementation of the pivot move in the general case, multidimensional rotations are required. The specific rotation matrix 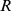 to apply can be computed from vectors 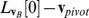 and 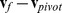. Even if 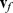 is properly chosen, the corresponding pivot move could be unfeasible because 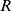 would map some residues onto positions that are either already occupied by the opposite branch (i.e. a clash occurs), or do not actually correspond to lattice vertices (in this case the elements of 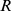 are not all integers). Anyway, the application of a pivot move in any case requires a significant computational effort [42], hampering its extensive employment in Monte Carlo methods.

Local moves are an effective means to explore the neighborhood of a given conformation, but in optimization problems like protein folding it is very difficult to edge away from local minima by their application in a random way. On the contrary, the pivot move may provide very significant conformation changes, but the more compact the conformation is, the more often the move comes to be unfeasible. A good tradeoff has been found with *pull moves*, originally introduced for square lattices [43], but also generalized to honeycomb [23] and 3D triangular lattices [44]. They exhibit the *semi-local property*, i.e., although in the worst case a pull move may relocate a large number of elements, on average only a few residues are involved (as witnessed also in experiments on FCC lattices by Jiang et al. [45]).

**Figure 7:**
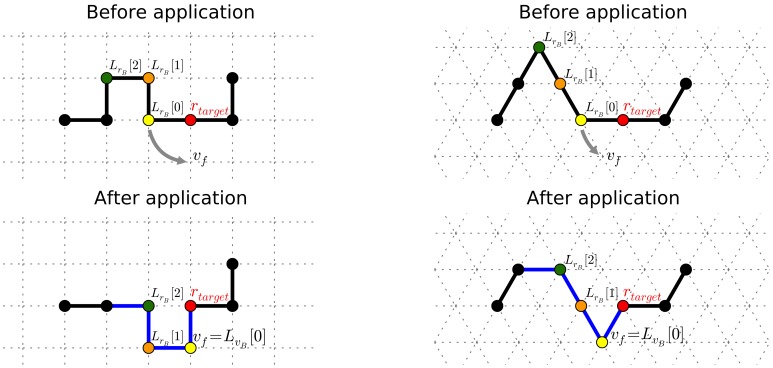
Examples of a pull move in 2D square (drawings on the left) and triangular (drawings on the right) lattices. The notation to indicate the involved elements is described in the text. The target residue is depicted in red, and the residues that undergo a displacement are colored. In the resulting configurations, the chain portion affected by the move is indicated in blue.

The description of the generalized **pull move** can make use of a notation similar to that already used for the pivot move. Figure 7 shows the application of a pull move on 2D square and triangular lattices and, despite its simpleness, can be useful to identify the involved elements.

One residue 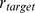 on vertex 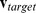 is initially selected, as well as one branch departing from it, either the forward or the backward one (it is the branch to be “pulled”). 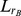 and 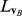 indicate respectively the lists of residues and absolute positions of the chosen branch, so that 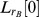 and 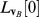 would correspond to the residue/vertex adjacent to 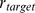 and 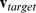, and so on. The symbol 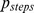 corresponds to the lowest number of steps the chain can be pulled in the given lattice type; it is topology-dependent, and its value is 2 for square lattices, and 1 for triangular ones.

The move can be specified by indicating what particular lattice vertex 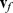, that is free, adjacent to 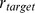, and 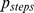 away from 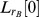, 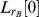 should be moved to. For the move to be feasible, there must exist a path from 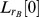 to 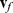 with 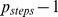 free internal vertices. Once 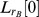 has been placed onto 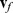, the branch must be pulled. Starting from 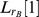 until the current residue does not need to be moved (or the list is finished), one residue 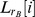 at a time is inspected and, in case it is not already adjacent to the previous one in the branch (i.e., 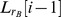), it is “pulled” back 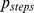 to become adjacent. Other graphical explanations of particular instances of this procedure are reported in previous works (among the others, [16], [39], [43], [44]).

From a software architecture standpoint, moves can correspond to methods of the Latticemodel class, and they operate on the configuration of any class instance. Also in this case, their code can be written in a lattice-agnostic way because the previous generalized definitions of moves are expressed in terms of adjacency and distances that, as already discussed, can be checked/calculated taking the lattice specifications as parameters. In Figure 4 only one method to perform a move is shown (namely, move_pullmove()). Moreover, at the class level it is convenient to envisage general move management methods, e.g., make_random_move(), along with setter/getter methods for parameters that determine the management strategies.

### Software

The described approach in dealing with general lattice models has been applied in an actual implementation in Python. Such a language has been chosen because it allows a quick and handy manipulation of the code, thus encouraging experimentations.

In investigations, performance was not the prime interest, basically because the main focus was on generic lattice modeling issues, and not on new efficient optimization methods. In any case, in the last years it has been shown that Python is also suitablefor challenging scientific computing [46], and for Bioinformatics problems as well [47]. The use of the numerical library *Numpy* [30], mainly for managing the representations of lattice vertices and configurations via arrays (not primitively supported by Python), let us obtain a reasonable performance level. Performance improvements can be obtained with more efficient compiled languages. The described software organization is suitable to be easily coded in any object-oriented language, such as C++.

All the tests presented in this paper have been run on an ordinary Mac notebook, with Mac OS ver. 10.6.8 on an Intel Core 2 Duo 2.4 GHz (but all the implementations are sequential ones), 2Gb RAM. Python ver. 2.6 and Numpy 1.6.2 have been used.

## Results and Discussion

The proposed general model has been defined first, and then its software implementation has been designed according to an object-oriented organization. Moreover, it has been stressed the need of a lattice-agnostic approach in shaping algorithms that operate on model instances. The primary result can be identified in the availability of a handy tool to explore the conformation space of lattice proteins, where the lattice model can be easily specified in a parametric way. At this point, it is crucial to better understand how the lattice type and the dimension affect the outcomes of the employed algorithms (also in terms of runtime), and how our tool can be used to get insights on specific models, or on comparisons among different ones. Some direct experiences with different kinds of optimization techniques are reported, in order to understand how the *generalized* algorithms, previously known in the literature only for basic cases, actually behave across the different models. Again, the main interest is not in developing new and better folding algorithms, but instead in investigating benefits and limitations of the whole tool as a means to explore the configuration space.

Two different classical approaches for the implementation of two different SpecificOptimizers (developed according to the pattern in Figure 5) have been considered: *chain growth* and *simulated annealing*. The former builds up the solution step by step, while the latter applies successive modifications to an initial conformation. In both cases, the classic HP potential has been chosen. The advantage of using these different algorithms in the present discussion is twofold: on one hand the main characteristics of the models can be uncovered, and on the other hand the flexibility of the model software implementation can be practically assessed. Details on the benchmark sequences used in the experiments are reported in Table 2.

**Table 2:** Benchmark sequences used in tests.

ID	Sequence	Len	#H	Notes
HI 1	HPH_2_PH_4_PH_3_P_2_HPHPHPHPHPHPHP_8_H	48	24	Harvard Instance [52]
HI 2	H_4_PHPH_5_PHPHPHP_6_HPHPHPHPHPH	48	24	Harvard Instance
HI 3	PHPHPH_6_PHPHPHPHPHPHPHPHPHPHPHPHP	48	24	Harvard Instance
HI 4	PHPHPHPHPHPHPH_5_PHPHPHPHP_4_HPHPHP	48	24	Harvard Instance
HI 5	PHPHPH_4_PH_4_PHPHPHPHPHPHP_6_HPH_2_PH	48	24	Harvard Instance
HI 6	HPHPHPHPHPHPHP_7_HPHPHPHPH_6_PH	48	24	Harvard Instance
HI 7	PHP_4_HPHPHPH_4_PHPHPHPHPHPHPHPH	48	24	Harvard Instance
HI 8	PHPHPH_4_PHP_6_HPHPHPHPHPHPHPHP	48	24	Harvard Instance
HI 9	PHPHP_4_HPHPHPHPH_6_PHPHPHPHPHPH_3_P_4_H	48	24	Harvard Instance
HI 10	PHP_6_HPHPHPHPHPHPHPHPH_7_PH	48	24	Harvard Instance
HI 9/2	PHPHP_4_HPHPHPHPH_6_P	24	12	First half of H 9
F 90	PHPHP_4_HPH_6_PHPHPHPH_5_PHPHPH_4_PHP_4_HPHPHPHPHPH PHPHP_4_HPHP	90	50	Seq. F 90_1 in [54]
S 1	H_4_PH_6_PHPH_8_PHPH_10_PHP_5_HPH_8_PHPHP_3_H_8_PH_6_PH_7_PHPH_9_P HPH_7_PHPH_7_PH_4_	135	100	Seq. S 1 - ditto
S 4	H_8_PHPH_3_PH_5_PH_6_PHPHPHPHPHPH_7_PHPHPH_6_PHPHPHP	164	100	Seq. S 4 - ditto
R 1	P_8_HPHPHPHPHPHPHPHP_4_H_6_PHP_5_HPHP	200	100	Seq. R 1 - ditto

Characteristics of the benchmark sequences used in the experimentation. The subscripts in sequence characters indicate the number of repetitions for each of them.

### Insights from Build-up Methods

*Chain-growth algorithms* represent a group of simple yet significant methods for protein folding in a lattice model [48]. Regardless of their specific details, all of them progressively build up the complete conformation by subsequent additions of chain chunks, and each chunk is placed in a way to minimize the potential of the protein aggregate built so far. At each step, an exhaustive exploration of possible placements is done for the next 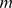 residues (so 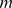 is named the *look-ahead parameter*), and the first 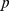 of them, 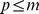, are used to build the next aggregate. Although alternative stochastic choices might be sensible [48], in the implementation presented here (called CgOptimizer) the target chunk conformation is always selected out of those that yield the current minimal potential, and this last choice takes into account only the chunks placed more closely to the current aggregate. According to the experiments, this strategy provides nearly-optimum conformations in a limited number of runs. Beyond the case of the chosen algorithm, the chain growth principle has been successfully applied also in other popular swarm intelligence methods to find ground conformations, e.g., in *ant colony optimization* (ACO), but only in two- and three-dimensional cases [40], [49], [50]. Moreover PERM [51], one of the most effective folding algorithms in the HP model, can be regarded as a particular form of Monte Carlo chain-growth.

It is worth noticing that the implementation of chain growth algorithms can be easily made lattice-agnostic by generically referring to the elements in main_dirs for the construction of the possible chunks to be added. Moreover, the correct definition of the potential to be used is directly available through the ordinary potential() method provided by the class Latticeprot.

**Figure 8:**
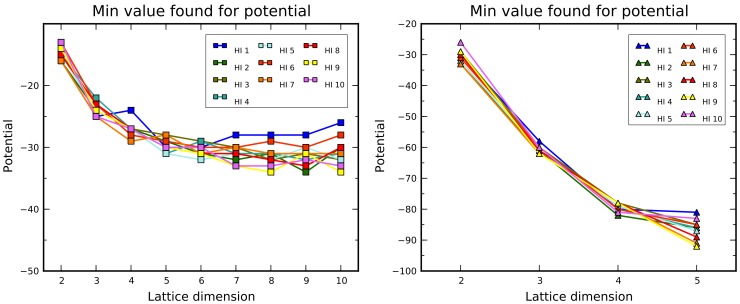
Increasing the lattice dimension, the chain growth algorithm finds protein configurations with progressively lower minimum potential. This holds for both lattice types (square on the left, triangle on the right).

Although CgOptimizer does not guarantee to find the optimal solution, it can obtain a decent lower bound just by running it a few times (at least for ordinary sequences). This procedure can be applied over different protein sequences, on different lattice types, and across multiple dimensions (reasonably only within the admissible range as defined in the first section). Results of this kind are shown in Figure 8. The benchmark sequences are the ten so-called “Harvard Instances” (HI), widely used for testing of folding algorithms since 1995 [52]. In the experiment, each test consists of ten optimization runs, executed on each sequence on both lattice types, on dimensions from 2 up to 10 for square lattices, and from 2 up to 5 for triangular lattices. Both the 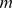 and 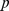 parameters have been set to 3. In the charts of Figure 7 the minimum values for the potential are shown, and each line connects the values for the same sequence at increasing dimensions.

The most evident feature is the decrease of the minimum potential for increasing dimensions, as sensibly expected. Keeping the same sequence, such a decrease becomes less pronounced at higher dimensions, and its trend looks to come to a sort of saturation. This phenomenon is not very manifest in the triangular case, because the lack of the parity constraint allows better accommodations of H residues, moving forward the beginning of saturation.

It is obvious that the theoretical trends in Figure 8 must be nonincreasing monotonic. Somehow surprisingly, in the square case for several sequences CgOptimizer has not been able to find better (or even equal) solutions than those found for lower dimensions. About this point, it must be noticed that i) on each test, because of long durations in the most demanding cases, the number of runs is necessarily limited and likely not sufficient to effectively sample the conformation space in higher dimensions, and ii) the look-ahead parameter 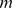 has been set to 3 to avoid excessive runtimes at high dimensions, but this hampers an effective handling of long “all-P” subsequences.

**Figure 9:**
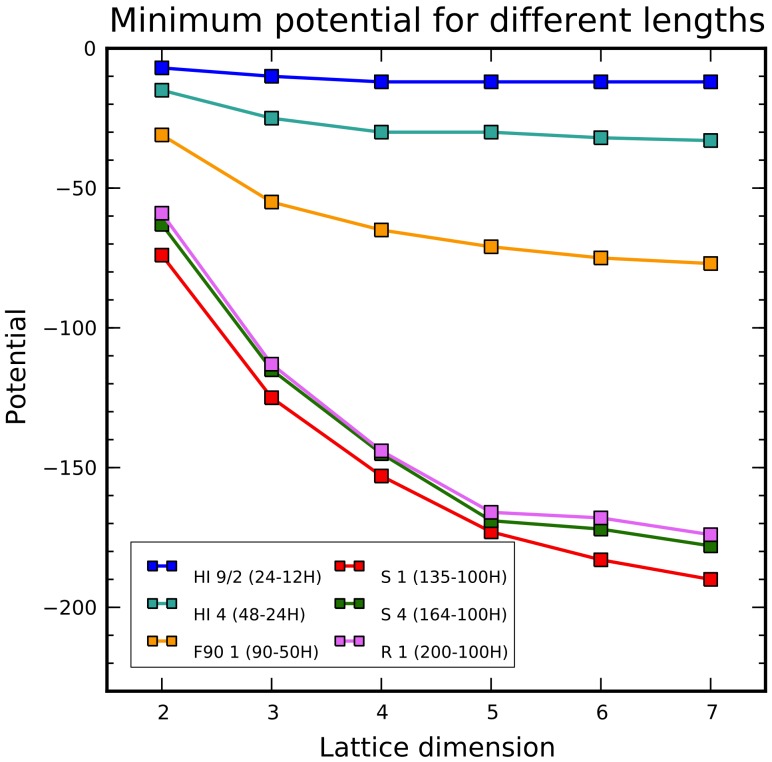
Experimental evaluation (by CgOptimizer) of the trend of minimum potential vs. dimension for different sequences on square lattices. In the legend, for each sequence, both length and number of H residues are specified.

The trend of minimum potential versus dimension deserves additional discussion. Beyond the simple experiment described so far, in order to empirically investigate the dependence on sequence length and characteristics, the chain growth optimization has been applied to multiple, heterogeneous sequences and the results are summarized in Figure 9. Usually longer sequences hit deeper minima and, the lower the minimum, the higher the dimension the trend saturation tends to occur. Actually, important factors are the number of H residues and the H/P ratio. The last three sequences in Figure 9 have different lengths but the same number of Hs, and the lower curve corresponds to the one with the highest H/P ratio.

**Figure 10:**
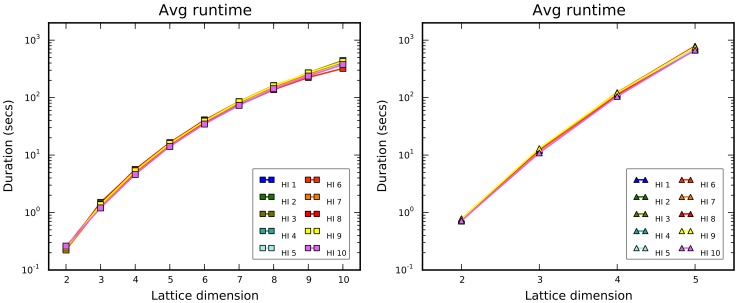
Average runtime for the chain growth algorithm. It is recorded for increasing dimension and for square (left) and triangle (right) lattices.

The analysis of runtime (Figure 10) is not particularly surprising, because the curves correspond to the temporal computational complexity for the algorithm. Specifically, it is 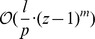, with 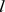 as the sequence length, 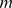 and 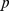 the chain growth parameters, and 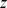 the lattice coordination number which in turn depends linearly on the dimension in the square case, and quadratically in the triangular one. To make Figure 10 more comprehensible, logarithmic scales have been used in the charts.

**Figure 11:**
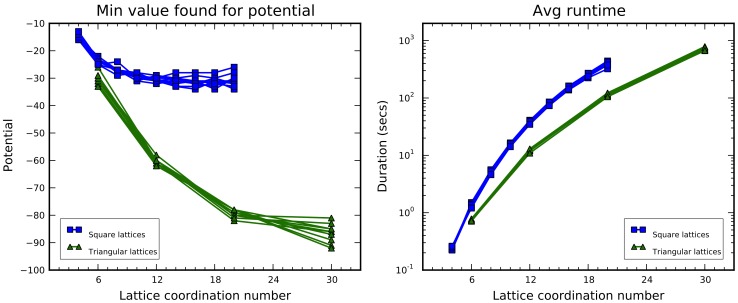
Comparison of the chain growth algorithm outcomes for square and triangle lattices, referring to the corresponding coordination numbers. The tests have been carried out over the HI sequences; the minimum potential value is shown on the left, and the runtime on the right.

A more objective comparison between results obtained for square and triangular lattices can be carried out focusing on the coordination number of the models. In fact, it would be interesting to check how in the general case models behave as the neighborhood varies. This can be done looking at Figure 11, which basically shows the same data of the previous charts organized in two single diagrams according to the corresponding values of the coordination number. Regarding the minimum potential, it becomes evident that the triangular lattice, due to looser topological constraints, is able to accommodate more hydrophobic residues around an H vertex respect to a square lattice with the same neighborhood value. Moreover, for the length of HI sequences, the potential looks to saturate within the admissible range in the square case, but not for triangular lattices. Regarding runtime (right chart in Figure 11), their values are very similar for both lattice types, and their trend over dimension is almost the same. For our implementation, triangular lattices are dealt with slightly more efficiently than the corresponding square ones. Finally, no significant difference in behavior can be noticed across the HI sequences. In the next tests, just HI 4 and the first half of H 9 (indicated as HI 9/2) are taken out of them as representative samples.

### Insights on Manipulation of Conformations

The examples seen so far do not consider the modification of conformations. To test also this aspect, an algorithm that makes use of *moves* must be taken as reference. Several Monte Carlo procedures have been developed for folding in the HP model, such as e.g. Evolutionary Monte Carlo [33], Monte Carlo Replica Exchange [39], and genetic algorithms [44]. Quantum annealing has been used for this purpose as well [53]. Moreover, some of the most effective folding algorithms recently designed employ moves in local optimizations within a larger hybrid approach [54].

One of the simplest Monte Carlo methods to find reasonable potential minima is the *simulated annealing* heuristic. Although its original formulation has shown to be scarcely effective in the exploration of protein conformation space (an early significant example for specific protein models is reported by Brower et al. [55]), particular versions have been employed also recently with good results on classical HP models on 2D square lattices [56] and 3D cubic lattices as well [57]. A simple implementation of simulated annealing will be taken as reference, to show issues in using move sets across different dimensions and lattice types.

In simulated annealing, the basic run (or *session*) is controlled along with the decreasing of a “temperature” parameter 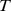 from a value 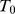 down to a final 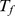. Starting from an initial conformation, at each temperature value a given number of steps 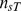 is performed. Each step consists of the application of a move to obtain a neighbor conformation, which may represent a better solution and may be possibly accepted as the current one. The acceptance is decided in the so-called Metropolis check, that depends on both the found potential difference 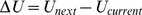 and the current value of 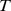: If 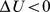 it is always accepted, otherwise it is accepted only with a probability given by the “Boltzmann” factor 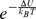 (usually 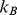 is taken as 1). The session outcome is the best conformation encountered throughout the whole procedure.

In addition to this general scheme, the used implementation in a class named SaSimpleOptimizer is characterized by the following details: i) an exponentially decreasing cooling schedule is chosen (i.e., 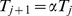 with 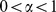); ii) as long as a session has been able to improve the solution (i.e., it has been *fruitful*), a successive one is performed, starting with the previous best conformation; iii) the initial conformation for the first session is a simple linear accommodation of the residues; iv) during the first half of the temperature range for the first session, referring to the generic formulation in eq. 9 the used potential holds 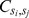 exactly as in the HP potential, and 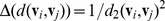. This choice is aimed at getting faster towards a globular conformation; v) no heuristic has been used in selecting the specific next move to apply (as it often happens in the most effective variants of simulated annealing). It has been shown in the literature [54] that properly modified fitness functions, as in iv) above, can dramatically improve the effectiveness of local searches.

The implementation of simulated annealing, as well as any other Monte Carlo algorithm that makes use of configuration modifications, is *automatically* lattice-agnostic because, in performing moves, it simply exploits methods that have been already written in a generic way as integral members of the class Latticemodel (see Figure 5). Multiple configurations for the same molecule can be kept in different copies of pos_abs.

The presented tests have been performed on a benchmark that include the sequences used for Figure 8, characterized by different lengths. For triangular lattices the last two sequences have been excluded, because of the excessively long runtime. For the same reason, each test on each sequence has been run only ten times, for both lattice types.

The values used for the simulated annealing parameters are the following, using the notation introduced above: 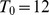, 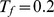, 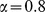, 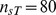, and 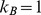. They have been chosen to produce a good solution in the basic case of a 2D square lattice in no more than two sessions. As the configuration space gets wider, reasonably an increasingly larger number of restarts should be needed. In this context, across different dimensions and lattice types, the number of fruitful sessions can give us a rough idea of the effort required by the Monte Carlo procedure (relative to the basic case) to single out a solution.

The used move set contain all the types discussed in this paper, implemented according to their general definitions. The random attempts to use move types obey a distribution with the following values of *try rate* 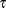 for all the move types: Slithering snake 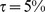, Pivot 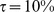, End 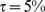, Kinkjump 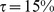, Crankshaft 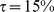, Pull 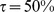. In particular, the value for the try rate of the pull move has been selected following the discussion reported by Thachuk et al. [39] (they use the symbol 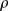 to indicate it).

**Figure 12:**
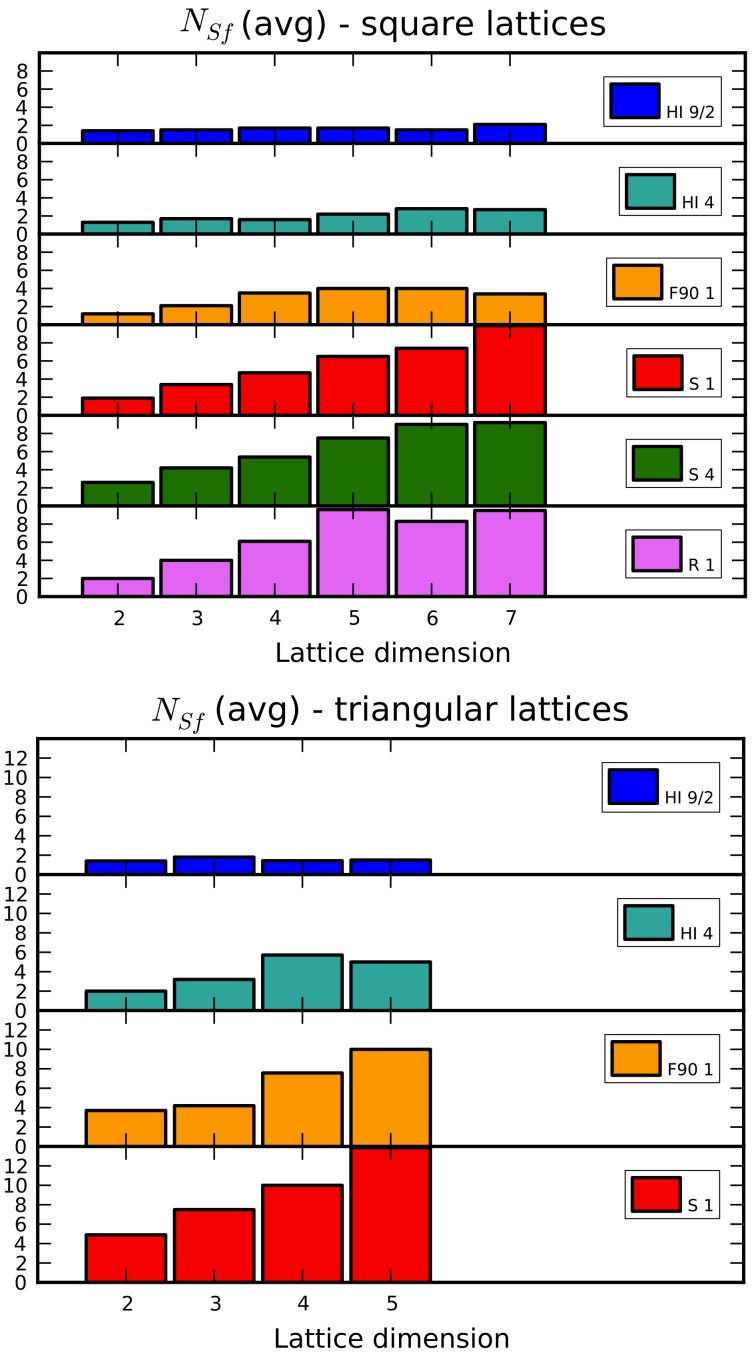
Number of sessions of simulated annealing that lead to a solution improvement. Results for both square and triangular lattices are reported.

A first point around the test outcomes is the evaluation of the average number of fruitful sessions 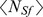; such results are reported in the charts of Figure 12. Informally, the value of this metric should grow as the sampling space widens out, and this may happen either increasing the sequence length, or the lattice coordination number 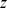. Figure 12 clearly shows that, regardless of dimension and lattice type, the longer the sequence, the larger 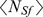, in accordance with our observation. Moreover, keeping the same sequence and increasing the dimension, 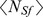 increases as well. Also for Comparing square and triangular lattices, the number of required restarts is similar when the lattice coordination number is the same.

**Figure 13:**
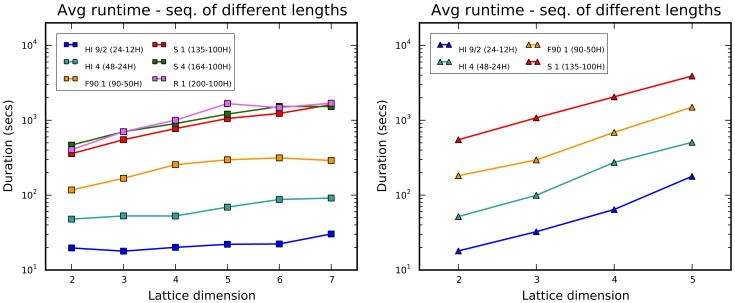
Average runtime for the simulated annealing optimization. It is recorded for the benchmark sequences for square (left) and triangular (right) lattices.

Although 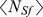 can quantify the effort in locating the solution, this metric does not reflect the overall computational weight of the procedure, because a single session usually asks for more operations as the sequence length and the dimension increases. An account of the average runtime for square and triangular lattices is presented in the charts of Figure 13. Although simulated annealing scales sufficiently well with dimension, its application beyond the admissible range (as defined in the first section) and with longer sequences becomes unpractical.

**Figure 14:**
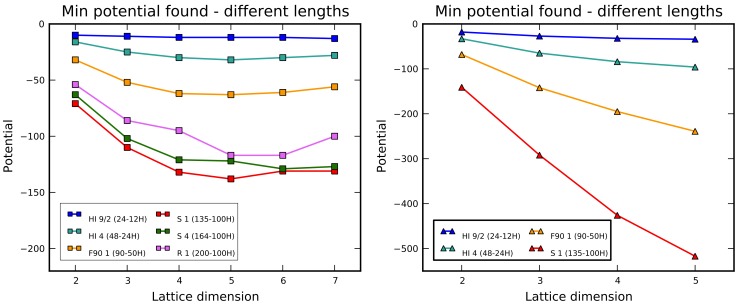
Minimum values for the potential, as found in the tests with simulated annealing for square (left chart) and triangular (right chart) lattices.

In order to assess the ability of our Monte Carlo procedure to find good optimal solutions, minimum potentials obtained by simulated annealing (see Figure 14) can be compared with those found by chain growth, that may be kept as decent approximations of the actual minima. Just after a quick look at the first chart of Figure 14 and Figure 9, it becomes evident at higher dimensions that the tests with simulated annealing provided worse approximations, and also the curves for each sequence in the square lattices are not monotonic decreasing. This aspect can be quantified by means of the relative variation of the value from simulated annealing respect to the corresponding one from chain growth, calculated as 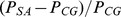 and reported in Tables 3 and 4. Somehow unexpectedly, for short sequences at the lower dimensions, simulated annealing behaves better than chain growth (upper left corner of the tables). On the contrary, as the sampling space gets wider (lower right corner), the solutions are in percent less and less accurate. Simulated annealing reveals its weakness in locating optima in these conditions, at least using the same parameters that turned to be effective for more limited explorations. In any case, a better effectiveness is experienced on triangular lattices.

**Table 3:** Potentials found: SA vs. CG (square lattices).

	Dim 2	Dim 3	Dim 4	Dim 5	Dim 6	Dim 7
HI 9/2	42.9	10.0	0.0	0.0	0.0	8.3
HI 4	6.7	0.0	0.0	6.7	−6.2	−15.2
F90 1	3.2	−5.5	−4.6	−15.5	−18.7	−27.3
S 1	−4.1	−12.0	−13.7	−20.2	−28.4	−31.1
S 4	0.0	−11.3	−16.6	−27.8	−25.0	−28.7
R 1	−8.5	−24.8	−34.0	−31.9	−30.4	−42.5

Comparison of minimum potentials obtained with simulated annealing and chain growth optimizers, run on square lattices. The reported values (%) correspond to the relative variation of s.a. respect to c.g.

**Table 4:** Potentials found: SA vs. CG (triangular lattices).

	Dim 2	Dim3	Dim 4	Dim 5
HI 9/2	28.6	3.8	0.0	0.0
HI 4	6.5	4.8	5.0	9.1
F90 1	0.0	−1.4	−2.0	4.4
S 1	−2.8	−8.2	−7.0	−5.3

Relative variation of minimum potentials obtained with simulated annealing respect to the corresponding values from chain growth optimizers, in the case of triangular lattices.

The facet that likely deserves more attention is the characterization of the usage of moves. In particular, for the different cases it is relevant understanding whether a random application of a move of a given type exhibits the same success probability, and how the moves that lead to an improvement of the solution are distributed among all the move types. To this aim, the following metrics can be used:

**hit rate** *h* i.e., the number of move applications that could be actually done over the total number of tried random move applications

**improvement fraction** *σ*i.e., the portion of all *effective* move applications that belongs to a given move type (an effective move is one that determined an improvement of the current solution).

The values of 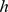 and 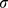 for our tests are reported in Tables 5 and 6. The slithering snake move in practice can always be feasibly applied for dimensions higher than 3, and its application is quite effective, considering that it is chosen only in the 5% of the move tries and that it holds a 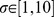. This move type is more effective at lower dimensions, and for square lattices.

**Table 5:** Characterization of the application of moves (square lattices).

		Dim 2		Dim 3		Dim 4		Dim 5		Dim 6		Dim 7	
		*h*	*σ*	*h*	*σ*	*h*	*σ*	*h*	*σ*	*h*	*σ*	*h*	*σ*
Sl. snake	HI 9/2	99	10	100	9	100	5	100	4	100	5	100	4
	HI 4	98	12	100	8	100	6	100	2	100	4	100	4
	F90 1	100	8	100	8	100	4	100	3	100	3	100	4
	S 1	89	10	93	6	96	4	100	2	100	3	100	2
	S 4	95	8	99	3	100	3	100	2	100	1	100	2
	R 1	100	10	100	6	100	3	100	3	100	3	100	1
Pivot	HI 9/2	59	4	73	3	81	4	87	5	92	13	92	11
	HI 4	49	7	59	5	68	7	73	7	81	11	86	14
	F90 1	40	8	44	4	52	4	66	11	71	9	78	11
	S 1	34	4	43	7	48	7	62	11	69	13	75	17
	S 4	34	7	44	9	51	10	67	14	69	18	83	23
	R 1	35	5	47	11	62	19	71	20	81	22	87	25
End	HI 9/2	80	0	96	0	100	1	100	0	100	1	100	0
	HI 4	80	0	96	1	100	0	100	0	100	0	100	0
	F90 1	88	0	99	0	100	0	100	0	100	0	100	0
	S 1	63	5	95	8	96	6	100	7	100	5	100	5
	S 4	66	5	98	3	100	4	100	3	100	3	100	3
	R 1	95	0	100	0	100	0	100	0	100	0	100	0
Kinkjump	HI 9/2	23	10	31	1	39	4	44	3	46	5	50	5
	HI 4	16	5	28	4	35	5	38	6	44	10	47	8
	F90 1	14	4	23	6	33	8	38	8	41	10	46	10
	S 1	11	5	22	8	29	9	35	9	39	11	41	11
	S 4	14	13	24	10	33	12	41	11	42	14	47	9
	R 1	16	5	29	9	38	11	43	10	45	12	48	9
Crankshaft	HI 9/2	14	0	47	6	62	11	73	9	77	11	82	16
	HI 4	11	0	41	12	61	12	68	19	76	19	80	18
	F90 1	8	2	34	10	52	22	64	22	68	26	77	23
	S 1	7	3	34	14	51	24	63	29	70	29	74	29
	S 4	8	4	34	17	52	23	68	31	70	29	77	29
	R 1	10	4	39	16	60	26	68	30	73	24	78	28
Pull	HI 9/2	44	77	92	81	100	74	100	78	100	65	100	64
	HI 4	38	75	82	69	98	70	100	66	100	56	100	56
	F90 1	32	78	75	73	96	62	100	57	100	53	100	52
	S 1	26	73	75	57	95	51	100	41	100	39	100	35
	S 4	30	62	75	58	96	48	100	38	100	35	100	35
	R 1	34	77	82	57	99	41	100	37	100	38	100	36

The outcome of the application of different types of moves depends both on the sequence and on the lattice dimension/type. Here, for the case of square lattices, are reported the hit ratio 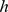 and the improvement fraction 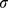 (both in %, with no decimals) for different sequences, in increasing length order.

**Table 6:** Characterization of the application of moves (triangular lattices).

		Dim 2		Dim 3		Dim 4		Dim 5	
		*h*	*σ*	*h*	*σ*	*h*	*σ*	*h*	*σ*
Sl. snake	HI 9/2	98	6	100	3	100	2	100	2
	HI 4	90	5	100	2	100	4	100	3
	F90 1	99	3	100	2	100	2	100	1
	S 1	67	2	100	2	100	1	100	1
Pivot	HI 9/2	61	6	70	11	78	15	83	12
	HI 4	53	14	57	11	62	11	70	12
	F90 1	48	11	49	11	60	10	62	12
	S 1	38	13	43	9	48	10	53	10
End	HI 9/2	94	0	100	0	100	0	100	0
	HI 4	86	0	100	0	100	0	100	0
	F90 1	96	0	100	0	100	0	100	0
	S 1	68	5	98	4	100	5	100	3
Kinkjump	HI 9/2	38	6	71	8	82	6	88	9
	HI 4	32	6	64	8	78	9	85	9
	F90 1	34	6	61	8	74	8	82	10
	S 1	26	7	52	10	71	12	76	12
Crankshaft	HI 9/2	29	14	79	9	94	14	97	15
	HI 4	25	9	72	14	90	15	96	10
	F90 1	27	9	66	10	89	17	94	16
	S 1	21	8	60	12	83	14	92	14
Pull	HI 9/2	70	67	96	69	100	63	100	62
	HI 4	66	66	93	65	99	62	100	66
	F90 1	67	71	90	68	99	63	100	61
	S 1	58	65	86	63	96	58	99	60

Here are reported the hit ratio 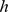 and the improvement fraction 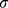 for different sequences in increasing length order on triangular lattices in dimensions from 2 to 5.

The pivot move increases its 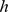 as the dimension increases, but usually decreases towards longer sequences. Its effectiveness varies considerably, but most of the times 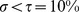.

The application of the end move is feasible almost all the times, but shows a very poor effectiveness.

The kinkjump and crankshaft moves increase their 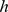 along with the lattice dimension. Crankshaft shows a better improvement fraction than kinkjump at higher dimensions.

Finally, the pull move (with 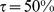) turns to be the most favorable type. It is very effective, and 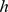 hits 100% in higher dimensions. The value of 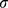 in one case reaches 81% (substantially larger than 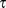), but usually decreases as dimension increases. In general, in triangular lattices 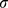 shows lower variations.

### Conclusions

So far, studies on simplified protein structures based on lattice models have directly targeted only a few specific lattice models. This paper has shown that it is possible to exploit a parametrical description of the underlying lattice, specifying its type and dimension. As a consequence, investigations can be conducted across different lattices simply by varying the proper parameters. Observing the neighborhood of alpha carbons in the core of real proteins, it can be easily noted that it is sensible making use of lattices whose dimension is larger than three. The proposed parametric models can automatically accommodate this possibility.

The theoretical tools for the proposed lattice models have been developed, shown and organized in the first part of the paper, indicating also how the basic concepts can be exploited in developing the supporting software. To this aim, the classical HP model has been considered. Moreover, an object-oriented architecture has been proposed for generic protein lattice models in any dimension, and a seamless hooking scheme for different optimizers has been suggested and implemented. The systematic adoption of such a software framework would make more meaningful and fair the comparison among different optimization methods.

The adoption of parametric models asks for lattice-agnostic implementations of all the algorithms that operate upon them. This issue has been explicitly addressed for the most important cases (e.g. for the calculation of the HP potential), suggesting to embed the basic functionalities directly in the protein model class. It has been necessary to provide general definitions for the move types proposed so far in the literature to modify protein configurations in Monte Carlo methods.

Experimentations with the proposed models have been carried out by means of a Python implementation, using sequences out of well established benchmarks widely studied in the literature. To point out the main features of the protein models varying lattice type and dimension, two simple yet significant classic optimization methods have been taken as references: Chain growth, and simulation annealing. It has been possible to experimentally uncover the trends of the minimum HP potential with increasing dimension for sequences of different lengths. Moreover, for the first time it has been shown a quantitative characterization of the employment and the effectiveness of move types usage in different lattice types and dimensions.

The shown theoretical and empirical results can be regarded as first steps in the investigation of multidimensional protein models and related algorithms. Some intrinsic geometrical features may restrict the application field of high-dimensional models. E.g., we can notice that in the N-dimensional continous space, the hypersphere surface/volume ratio is 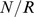, which can rapidly become very different from the “natural” 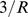 value as 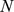 increases. Moreover, it is unclear whether multidimensional models may provide hints on the dynamic properties of protein systems. Further research is needed to ascertain the possible limitations of the general models.

It must be underlined that the simple optimizers used in the paper do not guarantee to find exact minima; for traditional models, the works by Backofen et al. [58], [59], based on constraint programming and a specific form of memo-ization, represent the reference approach but it reasonably would require an overall generalization in order to be applied to every possible case. Another example of non-trivial generalization to be carried out is the formulation of effective bounding functions in branch and bound algorithms for HP models [60], [61].

The proposed framework can be considered a novel general viewpoint in the study of protein lattice models, that subsumes several specific solutions proposed so far. The need to look at problems from a more general perspective may stimulate the reasoning on simplified models to better catch the basic characteristics of protein conformations and their manipulation.
